# Inferior ST-Segment Elevation Caused by Occlusion of Left Anterior Descending Artery

**DOI:** 10.25122/jml-2019-0079

**Published:** 2020

**Authors:** Hossein Sheibani, Amirhessam Kheirieh, Elham Azmoodeh

**Affiliations:** 1. Student Research Committee, School of Medicine, Shahroud University of Medical Sciences, Shahroud, Iran; 2. Vice-chancellery of Treatment, Shahroud University of Medical Science, Shahroud, Iran

**Keywords:** Myocardial infarction, LAD, Occlusion, Inferior leads ST-Segment elevation, Coronary angiography, Electrocardiogram

## Abstract

Here we present a case of a 70-year-old man with acute myocardial infarction caused by left anterior descending artery occlusion presenting as ST elevation in the inferior leads that suggested an occlusion of the right coronary artery. In contrast, coronary angiography results indicated a complete occlusion of the proximal left anterior descending coronary artery. We reported our observation in electrocardiographic data and coronary angiography and its changes after a percutaneous coronary intervention, and then we discuss its pathophysiologic mechanism.

## Introduction

On an electrocardiogram (ECG), ST-Segment elevation is the hallmark of myocardial infarction. An ECG showing acute ST-Segment elevations in the inferior leads suggests inferior wall infarction due to acute occlusion of the right coronary artery (RCA). However, acute occlusion of the left anterior descending (LAD) coronary artery that causes anterior wall infarction generally shows ST-Segment elevation in the precordial leads. Inferior ST-segment elevation caused by occlusion of LAD is rarely reported. In this paper, we describe a patient with ST-Segment elevation myocardial infarction (STEMI) that presented with ST-segment elevation in the inferior leads but with LAD occlusion, as revealed by his angiography results. This case is unique because LAD was not wrapped around and had no collaterals to the right coronary artery (RCA).

## Case report

**Figure 1:**
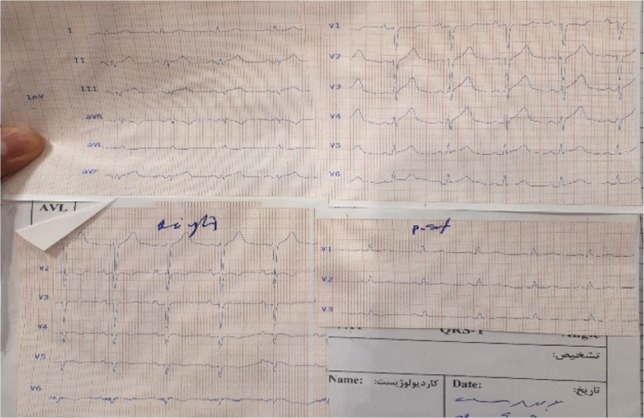
12-lead ECG (left, posterior and right leads) performed in our emergency room showing ST-segment elevation in the inferior leads (II, III, aVF) and V5, V6.

A 70-year-old man was referred to our hospital for sudden burning chest pain that radiated to his left shoulder and back for two hours, and it occurred immediately after exercise. He had no cold sweats, nausea, vomiting, and no dizziness. He was a nonsmoker but opium-addicted and had hypertension and hyperlipidemia diagnosed 15 years ago. Before this presentation, he underwent two angiographies and stent insertion. Upon physical examination, the patient showed no tachypnea, desaturation, cardiac murmur, abnormal breath sounds, or peripheral edema. The ECG performed in our emergency department demonstrated ST-segment elevation in the inferior leads (II, III, aVF) and V4-V6 (Figure 1). The first troponin in the emergency room was negative.

**Figure 2:**
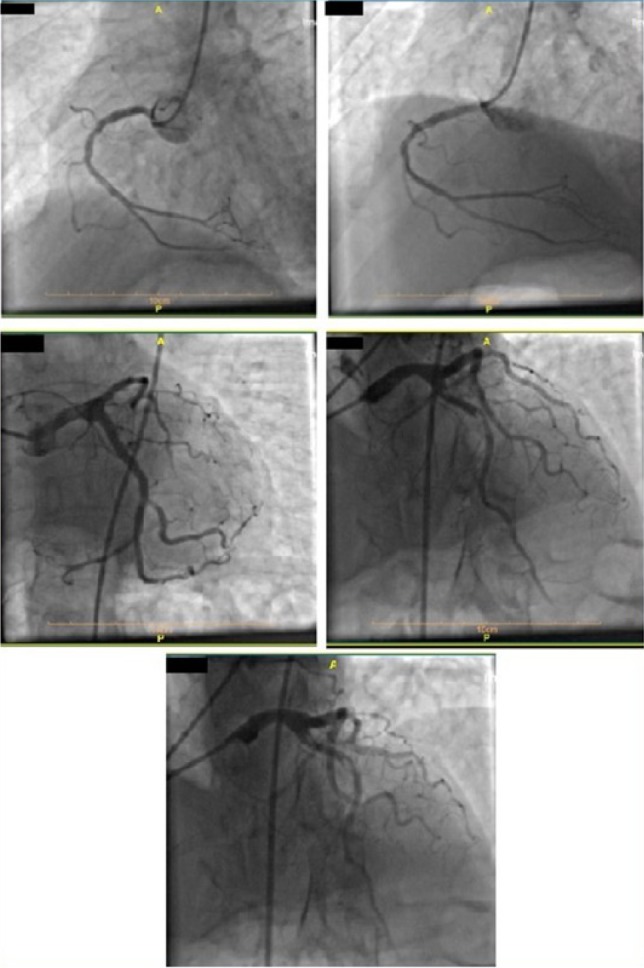
RCA with severe intimal irregularity and mild mid-part stenosis and good runoff. Significant stenosis due to stent thrombosis in the proximal part of LAD with distal delay, fair runoff.

After that, the patient received acetylsalicylic acid (300 mg) STAT, Plavix (300 mg) STAT, and loading doses of Atorvastatin (80 mg). We started glyceryl trinitrate by intravenous infusion immediately, and he was transferred to the cath lab within 30 minutes. Coronary angiography of the right superficial femoral artery (RSFA) showed near cut significant stenosis due to stent thrombosis in the proximal part of LAD with distal delay fair runoff (Figure 2).

**Figure 3:**
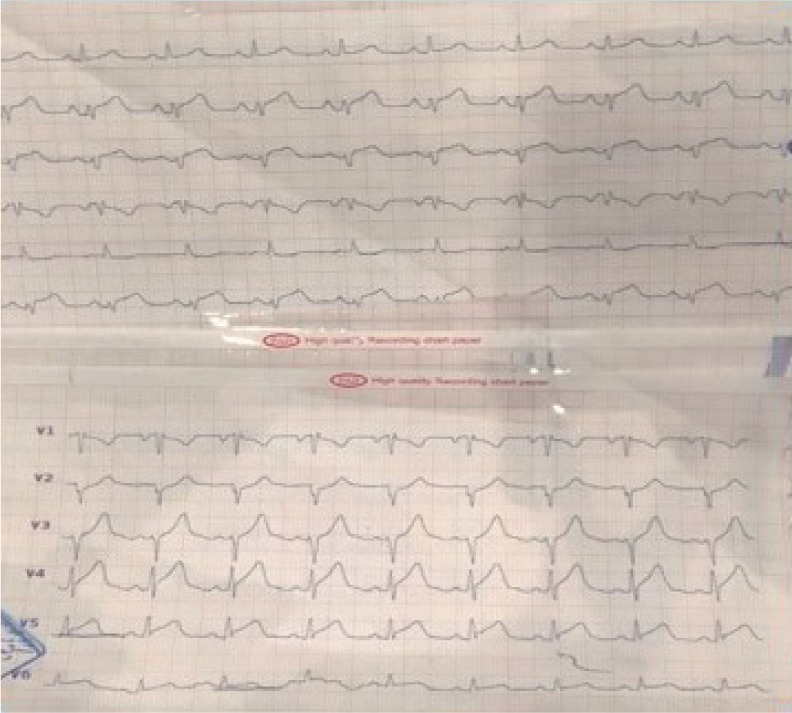
TTE results showed: mild LV enlargement with severe systolic dysfunction (EF: 25%). Akinesia in the inferior, inferolateral and anterolateral walls; the anterior wall is hypokinetic with normal thickness. Moderate Moderate diastolic dysfunction (grade 2), normal size and systolic function of the right ventricle. Mild mitral regurgitation, no mitral stenosis, trivial tricuspid regurgitation, insignificant pericardial effusion.

**Figure 4:**
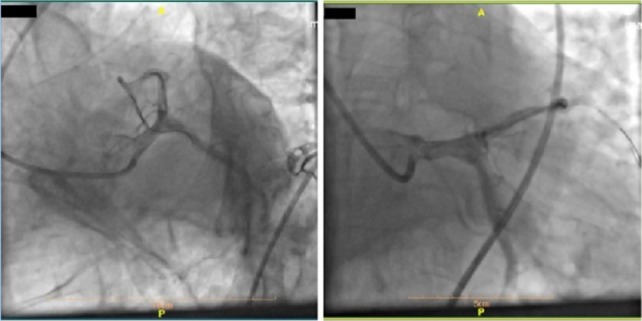
Wiring of the LAD artery.

**Figure 5:**
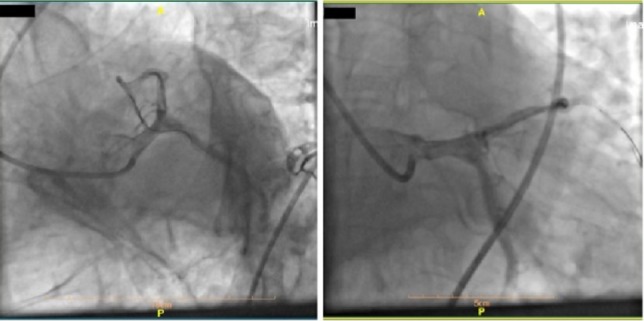
Stenting of the LAD artery.

**Figure 6:**
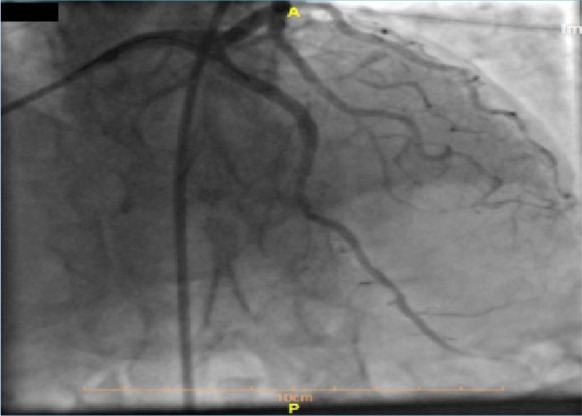
Final image with successful PCI of the LAD artery and good coronary flow.

**Figure 7:**
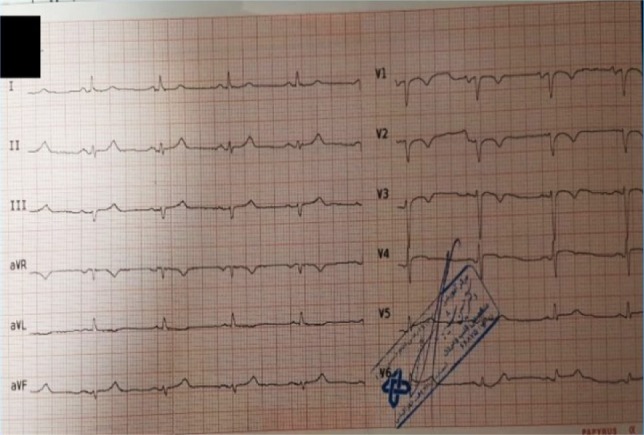
12-lead ECG after PCI; ST-Segment elevation returned close to baseline.

**Figure 8:**
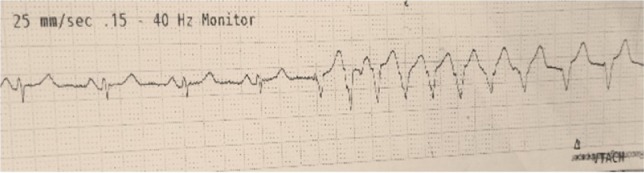
Non-sustained VT after reperfusion.

Transthoracic echocardiography (TTE) results before coronary artery bypass grafting showed an ejection fraction of 25% with significant regional wall motion abnormality, accompanied by akinesia in the inferior, inferolateral, and anterolateral walls as well as severe hypokinesia in the anterior and anteroseptal walls with a normal thickness (Figure 3). He underwent a percutaneous coronary intervention (PCI) on the proximal part of LAD with a drug-eluting stent that was successful with TIMI flow 3 throughout the LAD (Figures 4, 5, 6) and the patient was symptom-free (no chest pain and no arrhythmia) post catheterization and the ST-Segment elevation returned to near baseline (Figure 7). The patient had no significant arrhythmia after the reperfusion, except one episode of nonsustained ventricular tachycardia (VT) (Figure 8). After that, he received Atorvastatin 80 mg daily and Enoxaparin 60 mg BID (twice a day), being under constant surveillance. After that, the patient received acetylsalicylic acid 80 mg BID, Plavix 75 mg BID, Atorvastatin 40 mg QHS (every night at bedtime), Pantoprazole 40 mg daily, Aldactone 25 mg daily, Losartan 12.5 mg BID, Furosemide 20 mg daily, Alprazolam 0.5mg QHS, Carvedilol 3.125 mg BID. The patient stayed in the coronary care unit for three days and was discharged with a good general condition and stable vital signs.

## Discussion

Anomalous coronaries, depending upon the origin, course, and termination of the anomalous vessel, may be responsible for angina pectoris, heart failure, and sudden death [1]. Electrocardiography is still the gold standard for early diagnosing STEMI, and it can also reveal the location of the ischemia and infarct-related artery [2]. Acute occlusion of the RCA causes ST-Segment elevation on an ECG. On the other hand, an acute occlusion of the LAD that causes anterior wall infarction generally shows ST-Segment elevation in the precordial leads on an ECG. The LAD artery supplies the anteroseptal wall of the heart [3].

LAD lesions or occlusion are divided into proximal and distal groups [4]. Although ST-Segment elevation in the inferior leads when anterior myocardial infarction occurred due to LAD occlusion is rare, a study conducted by Bozbeyoğlu et al. showed that a patient with inferior ST-Segment elevation could have a wrap-around LAD more frequently than a patient without inferior ST-Segment elevation; also, distal occlusion of LAD is more frequent in patients with inferior ST-Segment elevation compared to patients without inferior ST-Segment elevation [5].

In a study conducted by Sapin et al., data indicated that the patient's ECG with wrapped LAD, which supplies the inferior wall of the left ventricle, showed ST-Segment elevation in the inferior leads during acute anterior myocardial infarction due to occlusion of the distal LAD artery [6].

Coronary artery anomalies are found in about 0.2 to 1.2 % in the general population, and most of them are discovered incidentally [6, 7]. Also, a wrap-around LAD cannot be reliably diagnosed by ECG [5]. According to Sapin's study, two conditions are considered to be essential for finding inferior ST elevation during anterior acute MI: 1) The mass of ischemic anterior wall myocardium is relatively small, resulting in a weaker anterior injury current and less reciprocal inferior ST-segment depression; and 2) there is concomitant inferior wall transmural ischemia that further shifts the inferior ST-segment upward. In this study, angiography was performed and coronary angiograms were examined for three features:

• Site of LAD artery occlusion (a distal obstruction implying a smaller mass of ischemic anterior wall myocardium,
• LAD artery extension onto the inferior wall of the left ventricle (wrap-around LAD)
• Collateral flow from the LAD artery to the inferior wall.

The latter two features would be expected to contribute to the inferior wall transmural ischemia [5].

**Figure 9:**
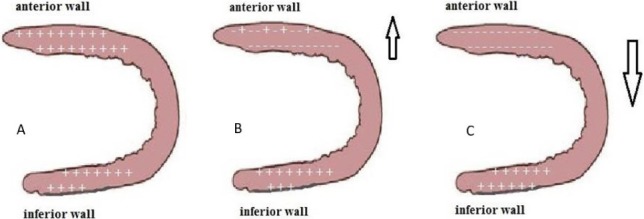
A. Normal electric potential of the left ventricle myocardium; B. Electric potential in case of anterior myocardial infarction. C. Electric potential in case of inferior myocardial infarction.

Another cause of ST-segment elevation was discovered recently, and it is acute LAD stent thrombosis following primary PCI for LAD occlusion, and it seen in the inferior leads. In normal myocardial tissue, the extracellular electrical potential is positive can be explained by electrical events (Figure 9A). In other words, the extracellular positive electrical potential in the anterior wall tends to be negative, owing to the injury from acute anterior MI (Figure 9B). Stent thrombosis after primary PCI for LAD rapidly impairs the perfusion of the anterior myocardial tissue, which is the reason for the negative electrical potential of the tissue under the injured front wall to become more pronounced. The extracellular potential of the inferior myocardial wall stays positive during MI, so this is the reason for ST-Segment elevation in the inferior leads that could be the vector that heads for negative to positive extracellular potential (Figure 9C) [5, 8]. However, interventional cardiologists and cardiac surgeons and even physicians should be updated and aware of such a rare anomaly as it can have a significant impact on the clinical outcome of a patient and can protect the myocardium from chronic ischemia.

## Conclusion

There are four features considered to be essential for finding inferior ST-segment elevation during anterior acute MI:

1. The site of LAD artery occlusion (distal occlusion).
2. LAD artery extension on the inferior wall of left ventricle (wrap-around LAD).
3. Collateral flow from the LAD artery to the inferior wall.
4. Acute LAD stent thrombosis following primary PCI of LAD occlusion.

In our case, there was an LAD occlusion presenting as ST-segment elevation in the inferior leads that suggested an occlusion of the right coronary artery on the ECG; however, coronary angiography results demonstrated a complete occlusion within the proximal part of LAD.

Therefore, early recognition and subsequent initiation of the appropriate management may change the outcome of the disease.

## Conflict of Interest

The authors confirm that there are no conflicts of interest.

## References

[1] Dubey L (2013). Anomalous origin of the coronary arteries: an account of six cases. Journal of the Nepal Medical Association.

[2] Slavich G, Poli S, Spedicato L, Sappa R, Trianni A (2012). Electrocardiographic identification of the culprit artery and occlusion site in ST-elevation myocardial infarction. Giornale italiano di cardiologia (2006).

[3] Brown KN, Borger J (2019). Anatomy, Thorax, Heart Anomalous Left Anterior Descending (LAD) Artery.

[4] Fujii T, Hasegawa M, Miyamoto J, Ikari Y (2019). Differences in initial electrocardiographic findings between ST-elevation myocardial infarction due to left main trunk and left anterior descending artery lesions. International journal of emergency medicine.

[5] Bozbeyoğlu E, Yıldırımtürk Ö, Aslanger E, Şimşek B, Karabay CY, Özveren O (2019). Is the inferior ST-segment elevation in anterior myocardial infarction reliable in prediction of wrap-around left anterior descending artery occlusion?. Anatolian journal of cardiology.

[6] Sapin PM, Musselman DR, Dehmer GJ, Cascio WE (1992). Implications of inferior ST-segment elevation accompanying anterior wall acute myocardial infarction for the angiographic morphology of the left anterior descending coronary artery morphology and site of occlusion. The American journal of cardiology.

[7] Click RL, Holmes DR, Vlietstra RE, Kosinski AS, Kronmal RA (1989). Anomalous coronary arteries: location, degree of atherosclerosis and effect on survival—a report from the Coronary Artery Surgery Study. Journal of the American College of Cardiology.

[8] Wagner GS (2001). Marriott's practical electrocardiography.

